# First person – Taisa Bohdanovych

**DOI:** 10.1242/bio.061955

**Published:** 2025-03-19

## Abstract

First Person is a series of interviews with the first authors of a selection of papers published in Biology Open, helping researchers promote themselves alongside their papers. Taisa Bohdanovych is first author on ‘ Comparison of silver and gold nanoparticles green synthesis by *Artemisia annua* hairy root extracts’, published in BiO. Taisa is a junior research scientist in the lab of Dr Nadiia Matvieieva at the Department of Genetic Engineering, Institute of Cell Biology and Genetic Engineering of the National Academy of Sciences of Ukraine, investigating antioxidant, anti-inflammatory and other bioactive compounds of plants and hairy root cultures.


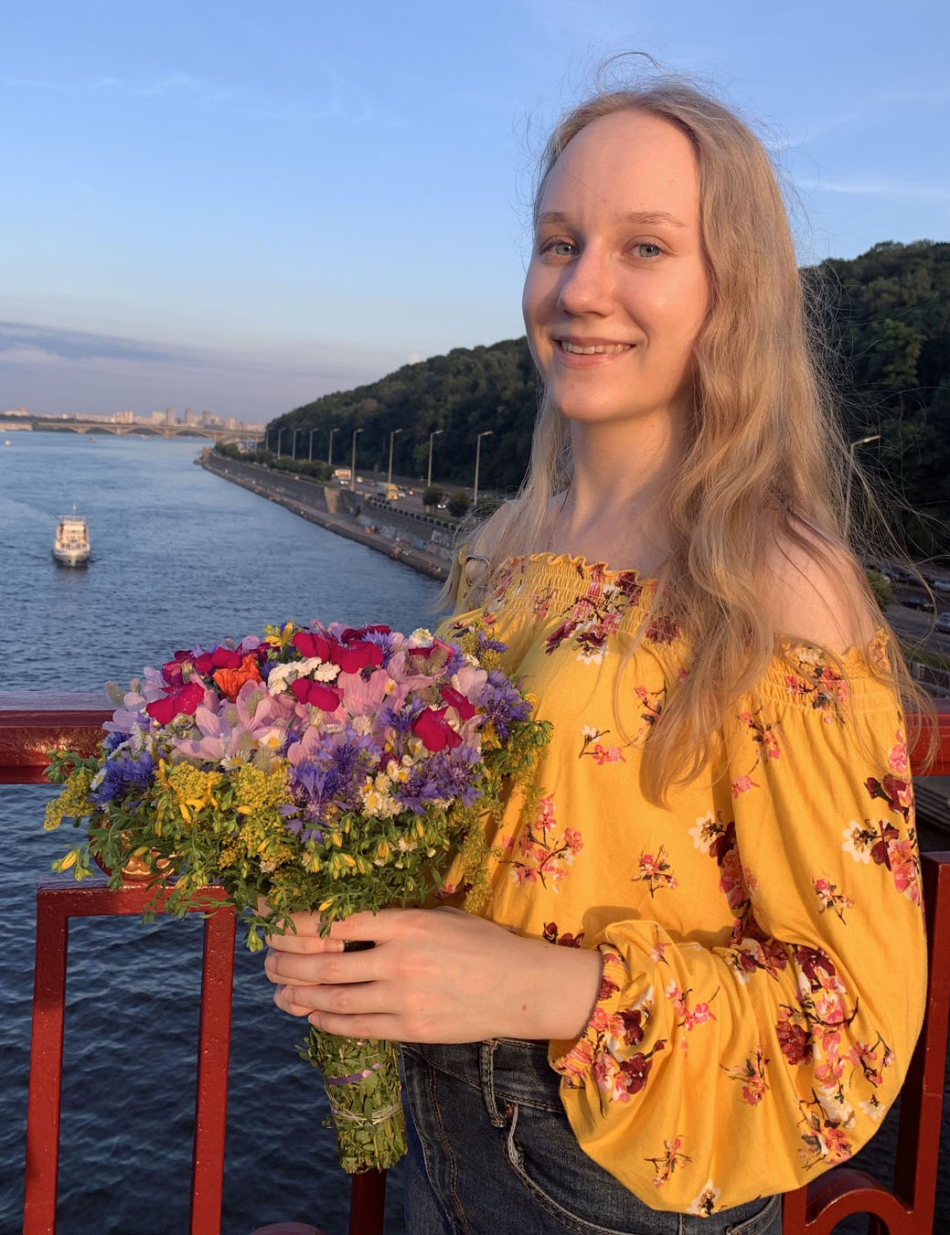


**Taisa Bohdanovych**

**Describe your scientific journey and your current research focus**

My journey in science and research started when I was a biotechnology student. During my time at university I have conducted mini-projects/practical studies in various areas of biology, starting from microbiology to pre-clinical research and R&D at a pharmaceutical company, to understand what I would like to do as a job. I realized that I want to work with plants and hairy roots as they are a boon of biopharmaceuticals. Thus, my PhD thesis was about the extracts of transformed root cultures (hairy roots) of Tilesius’ wormwood with boosted secondary metabolism and a variety of biological activities. Currently I work with hairy roots of other medicinal plants with focus on their reducing power.

**Who or what inspired you to become a scientist?**

Curiosity and eagerness to understand how the world works have always pushed me to become a researcher in natural sciences. It is fascinating to me that in each plant cell thousands of processes occur at any moment, and there is always something to study for each new generation of scientists. Research and altering of those metabolic pathways continue to result in new biopharmaceuticals that are of use for medicine, veterinary and industry.

This study is the first to compare the synthesis of nanoparticles of different metals using the same extracts from wormwood hairy roots.

**How would you explain the main finding of your paper?**

The study showed the potential and limitations of *Artemisia annua* hairy root extracts in the green synthesis of gold and silver nanoparticles with insights into their structural and functional differences. Such synthesis has many advantages over chemical and physical ones, avoiding the use of toxic agents that can pose a potential risk to environment. The differences in extract composition may have caused those variations in the number, shape, and activity of obtained nanoparticles.

**What are the potential implications of this finding for your field of research?**

This study is the first to compare the synthesis of nanoparticles of different metals using the same extracts from wormwood hairy roots. It provides evidence that the composition and concentration of the plant/hairy root extract may influence not only the characteristics of obtained nanoparticles, but the stability of the colloid solutions.

**Figure:**
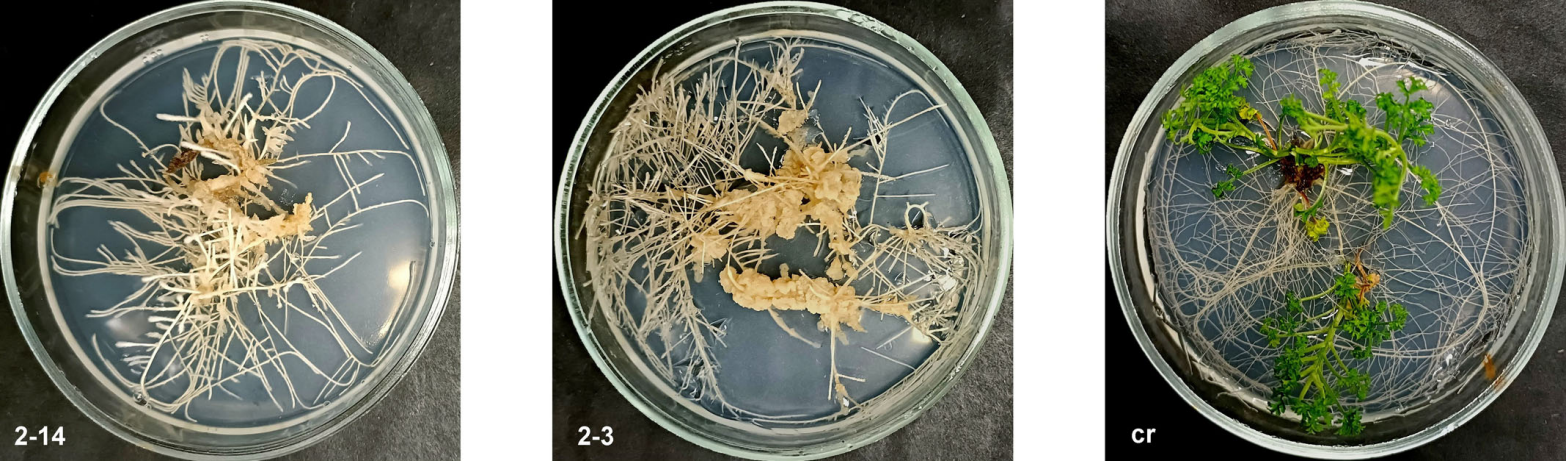
*Artemisia annua* L. control plants (cr) and hairy root lines (2-3 and 2-14).

**Which part of this research project was the most rewarding?**

This research project was the first collaboration of our laboratory (of adaptational biotechnology) and the center for collective use of scientific instruments/equipment, mass spectrometry and liquid chromatography, of the National Academy of Sciences of Ukraine (Chuiko Institute of Surface Chemistry of NAS of Ukraine) to perform HPLC and MALDI MS studies. We hope to continue joint projects in the nearest future. As well, I had a chance to try and optimise a new method for our laboratory – the photocatalysis of Methylene Blue solution by nanoparticles.

**What do you enjoy most about being an early-career researcher?**

I enjoy the possibility to continuously expand my research and explore new ideas. I constantly search for new projects, schools for early-career researchers and various courses to deepen my knowledge and be a better scientist.

Each problem usually has several solutions

**What piece of advice would you give to the next generation of researchers?**

Try to keep going even through frustration. Sometimes science can be very challenging, and the best you can do is to continue exploring new ideas, points of view, and methodologies. Each problem usually has several solutions, and it is our job as researchers to find the best and most optimal ones.

**What's next for you?**

Nowadays, the future of science in Ukraine is very uncertain, unfortunately. I plan to continue and expand my research in the fields of biopharmaceuticals and activity of medicinal plants the best I can.
